# A Computational Approach to Qualitative Analysis in Large Textual Datasets

**DOI:** 10.1371/journal.pone.0087908

**Published:** 2014-02-03

**Authors:** Michael S. Evans

**Affiliations:** Neukom Institute for Computational Science and Department of Film & Media Studies, Dartmouth College, Hanover, New Hampshire, United States of America; Université de Montréal, Canada

## Abstract

In this paper I introduce computational techniques to extend qualitative analysis into the study of large textual datasets. I demonstrate these techniques by using probabilistic topic modeling to analyze a broad sample of 14,952 documents published in major American newspapers from 1980 through 2012. I show how computational data mining techniques can identify and evaluate the significance of qualitatively distinct subjects of discussion across a wide range of public discourse. I also show how examining large textual datasets with computational methods can overcome methodological limitations of conventional qualitative methods, such as how to measure the impact of particular cases on broader discourse, how to validate substantive inferences from small samples of textual data, and how to determine if identified cases are part of a consistent temporal pattern.

## Introduction

Conventional qualitative methods for analyzing text are valuable, but they are also labor-intensive and do not scale well beyond small textual samples. Close reading and manual content coding, for example, are increasingly inadequate for examining the large textual datasets that are emerging as archives of books, newspapers, journal articles, and media transcripts assume digital form. In this paper I address this pressing methodological problem by demonstrating how computational methods can extend the reach of qualitative analysis into large textual datasets.

As an illustrative exercise I analyze demarcation between science and non-science in public discourse. Such demarcation is conventionally analyzed through close readings or qualitative coding of small textual datasets generated during specific cases of scientific controversy, for example dozens of personal letters [1], twenty-one interviews [2], or the publications of two participants in a dispute [3]. In this exercise I analyze demarcation in broader public discourse by applying probabilistic topic modeling techniques to thousands of documents published in American newspapers from 1980 through 2012.

Through this exercise I demonstrate how computational data mining techniques can identify, and evaluate the significance of, qualitative information in large textual datasets. I also advance qualitative methodology by introducing computational techniques for measuring the impact of particular cases on broader discourse, validating substantive inferences from case studies, and determining if identified cases recur as part of a consistent temporal pattern.

## Materials and Methods

### Dataset construction

For this exercise I constructed a large textual dataset from newspapers, which provide the “master forum” for public discourse [4]. The Lexis-Nexis Academic US Major Papers service to which my institution subscribes contains links to archived records from approximately 30 public newspapers of varying market sizes and geographical distribution in the United States. I used a keyword search to identify and retrieve all matching documents that had been published from 1980 through 2012. The historical cutoff date was selected based on preliminary ngram searches of large published corpora that showed a dramatic increase in demarcation language from 1980 onward.

**Figure 1:**
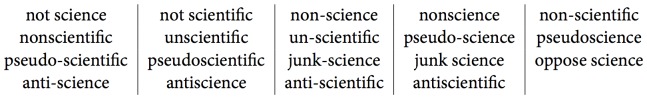
Dataset search keywords. Keyword phrases used fuzzy matching to additionally capture close but not identical phrase matches, such as “not really science.”

Figure 1 lists the keywords used to identify and retrieve documents. These keywords demarcate science by specifying what it is not. For example, an article might quote a source saying that creationism is “not science,” that astrology is “pseudo-science,” or that denying climate change is “anti-science.” Unlike case studies that focus on particular controversial subjects, here no subject constraints were placed on data collection. The sample for this exercise consists of thousands of documents from a broad range of public discourse rather than a small number of documents about a few subjects.

The search results were reviewed for technical validity, and approximately 2% of retrieved documents were excluded from the final dataset on technical grounds. Examples of technical exclusions include documents containing fewer than 200 characters, documents accidentally retrieved through systematic false positive matches (e.g. “U.N. scientific” rather than “unscientific”), documents that were not archived in a usable format (e.g. photo essays or statistical tables), and documents that were duplicated in the archive. The resulting master dataset contained 14,952 documents. Metadata for each document included date of publication and original source.

### Topic Discovery

Analyzing thousands of documents using conventional qualitative coding techniques is practically impossible. I employ computational methods for topic discovery to move beyond this limitation. Computational topic modeling methods have been used successfully to answer questions that might otherwise be addressed by qualitative coding methods, such as which areas of science are growing or shrinking [5], how the substantive contents of a colonial American newspaper changed over time [6], how members of Congress differ in their communications with constituents [7], and how styles of political communication relate to political polarization [8].

I apply a probabilistic topic modeling technique called Latent Dirichlet Allocation (hereafter LDA) [9]. Given a text corpus, LDA assumes that topics are latent patterns of words in the corpus, and calculates such topics as a probability distribution over words [10]. In the basic LDA model, any document can be described as a mixture of topics. So, for example, an LDA analysis of scientific abstracts might find one topic with the words “genetic embryo somatic dna” and another topic with the words “viral allograft antigen lupus.” The analyst can then apply topic labels to indicate that one topic is focused on *reproductive genetics* and the other topic is focused on *immunology*. LDA estimates the probability that “viral” will be associated with “viral allograft antigen lupus” (*immunology*), the probability that the topic (*immunology*) will show up in any document, and the exact mixture of the resulting topics for each document in the corpus (e.g. 75% *immunology*, 25% *reproductive genetics*). The result resembles qualitative human classification of subject matter, but is generated probabilistically through computation [11].

Because topic modeling is probabilistic, selecting an appropriate topic model involves a variety of tradeoffs and judgments by the human researcher. It is conventional to generate a range of candidate topic models, then use several qualitative and quantitative validation techniques to select the model that is the best fit for the specific research question [11]. For this exercise I started by generating nine candidate topic models with different numbers of possible topics ranging from 15 to 100 topics. All candidate topic models were generated using the MALLET software package with hyperparameter optimization enabled [12]. MALLET was chosen for its speed and memory advantage over alternatives, as it implements the SparseLDA algorithm [13]. Topic model data output from MALLET included top topic words and phrases, topic-specific Dirichlet parameters, word-topic counts, topic word weights, and document-level topic proportions.

### Model Selection

All candidate topic solutions were subjected to a validation process consisting of three phases. In the initial validation phase, I reviewed the top 50 terms generated for each topic in each model to determine intelligibility. The basic test was whether or not I could summarize each topic in a brief label, such as “religion” or “presidential politics.” I also flagged probable “junk topics” such as overly-broad topics consisting of common adjectives or general terms [14]. Most candidate models were rejected at this first phase. In the case of models with lower number of topics (15, 20, 30) the topics were overly broad and combined words from obviously different subjects into a single topic. For example, in the 20 topic model, one topic judged to be too broad contained top words such as “war people race jewish trade.” At the other extreme, topic models with higher number of topics (60, 75, 100) offered many additional identifiable topics, but these topics were often too specific, either geographically or conceptually, to be useful in the analysis. For example, in the 100 topic model, one topic judged to be too specific contained top words such as “minnesota weather snow weeks.”

In the second validation phase I used quantitative diagnostic data from MALLET to verify or reject initial qualitative analysis of topics. In additional to conventional output of topic model data, MALLET provided diagnostic information such as topic-specific distribution over words relative to corpus distribution [14] and topic coherence [15]. (MALLET diagnostic files were translated into Microsoft Excel-friendly formats using Perl and Python scripts provided in Andrew Goldstone's *dfr-analysis* GitHub archive at https://github.com/agoldst/dfr-analysis.) In ambiguous cases that had been flagged as suspicious in the first phase of validation, these quantitative features provided additional information for rejecting topics that were overly broad or internally incoherent. At this phase topic solutions of 40 and 50 topics were rejected, primarily based on having too many topics that initially appeared coherent but on further review proved either to combine different subjects into single topics or to divide an identifiable subject across multiple topics.

In the third validation phase I incorporated qualitative domain knowledge to “predict” various features of the remaining model and verify that these occurred as expected. I compared the relative distribution of topics over documents to verify that topics that might be expected to occur together more often in documents (e.g. topics focused on dining and cooking) had more similar distributions to each other than to topics that domain knowledge would suggest are unrelated (e.g. topics focused on cooking and space). I also charted the distribution over time for selected topics to verify that higher prominence in the corpus coincided with known events that should affect such distribution. For example, a topic that appears to be about presidential politics should be more prominent in presidential election years.

**Figure 2:**
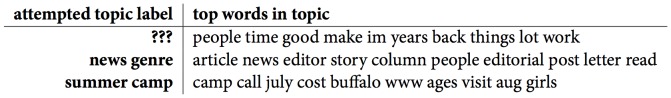
Examples of rejected candidate topics from 45 topic solution. Ten most likely words for each topic listed to right of attempted topic label.

The 45 topic solution emerged from the validation process as the best tradeoff between specificity of topics and significance of topics that still retained analytical interpretability. However, like any topic modeling solution, this solution still included some topics that were incoherent, not substantive, or very infrequently occurring [16]. Figure 2 provides examples of rejected candidate topics. The topic labeled “???” in Figure 2 provides an example of an incoherent topic consisting of various common words that occur in many documents but are not substantively related. An example of a topic that is coherent but non-substantive is the topic labeled “news genre,” which contains terms that are associated with journalistic writing style rather than any particular substantive content. Finally, an example of a coherent but very infrequently occurring topic is the topic labeled “summer camp,” which captures information about children's summer camps. I disregard in the analysis twelve topics from the 45 topic solution that are incoherent, non-substantive, or very infrequently occurring.

## Results and Discussion

### Identifying Substantive Topics

**Figure 3:**
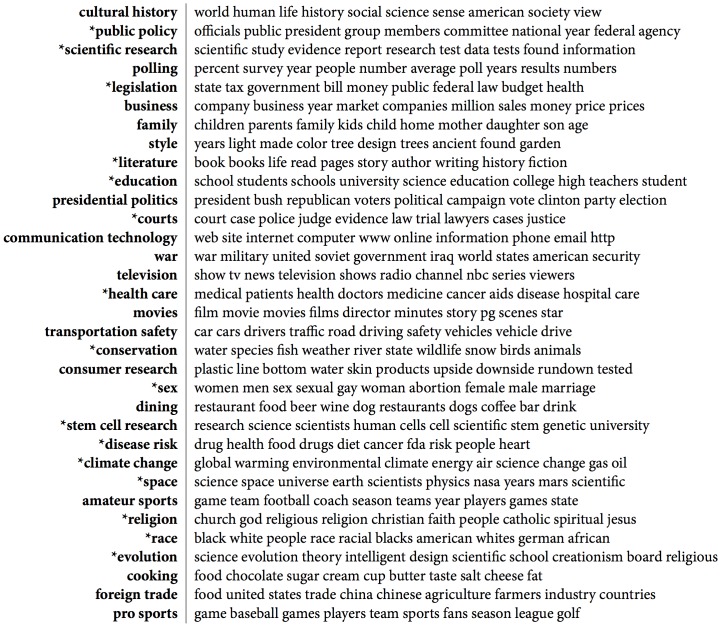
Labeled substantive topics. Ten most likely words for each topic listed to right of assigned topic label. Asterisks indicate subjects previously identified in qualitative case studies of demarcation.

After validation, 33 substantive topics emerged from within the 45 topic solution. Substantive topics are topics with word and phrase content that is focused on identifiable subjects of public discussion. For example, a topic containing words such as “show tv news television shows radio channel nbc series viewer” is probably focused on television. For each substantive topic I assigned a concise label to indicate the subject on which that topic's words and phrases are focused. I note these substantive topic labels in the remainder of the paper by using italics, for example *television*. Figure 3 reports substantive topics with their assigned labels and the top ten most likely words in each topic. Note that the specificity of a topic label reflects all of the words in the topic, which usually number in the hundreds of words, and not simply the top ten words shown here.

After assigning topic labels, I noted which topics in the model were focused on subjects that had already been identified in qualitative case studies of demarcation. In Figure 3 I indicate these topics using asterisks. Such topics include *education* [1], *literature* [17], *scientific research* [18], *public policy* [19], *evolution* [20], *stem cell research* [21], *race* [22], *religion* [23], *courts* [24], *legislation* [19], *disease risk* [25], *conservation* [26], *health care* [27], *sex* [28], *space* [29], and *climate change* [30].

At the document level, typical documents with high proportions of these topics have titles such as “Intelligent Design: Ruling Bans Discussion of Concept” (*evolution*), “Harvard Plans Center to Grow Stem Cells” (*stem cell research*), “Race Has No Basic Biologic Reality” (*race*), “Humans May Double the Risk of Heat Waves” (*climate change*), “Scandals Point to Weakness in Review Process” (*scientific research*), and “Vitamin C: Is Anyone Right On Dose?” (*disease risk*). Illustrative examples of demarcation language from documents with high proportions of these topics include:

- “... intelligent design ‘is a religious view, a mere re-labeling of creationism, and not a scientific theory’” (*evolution*)
- “It is very troubling to see a major public policy decision about medical research made from an unscientific point of view.” (*stem cell research*)
- “... differentiating species into biologically defined ‘races’ has proven meaningless and unscientific as a way of explaining variation, whether in intelligence or other traits.” (*race*)
- “‘Modeling is not science,’ said Ebell...” (*climate change*)
- “Thus, to many critics, peer review is a pseudo-scientific name given to an editorial process not unlike that common to many forms of journalism.” (*scientific research*)
- “'He had the best of intentions, but he did not have the science to support his hypothesis, Dr. Levine said.” (*disease risk*)

Computational methods can also identify previously unrecognized subjects of discussion in large textual datasets. As Figure 3 shows, in this exercise topic discovery also successfully identified many topics focused on subjects that had not been previously identified in qualitative case studies of demarcation. Such topics include *cultural history*, *cooking*, *presidential politics*, *movies*, *family*, *style*, *consumer research*, *polling*, *business*, *dining*, *communication technology*, *foreign trade*, *pro sports*, *war*, *television*, *transportation safety*, and *amateur sports*.

At the document level, typical documents with high proportions of these topics have titles such as “Consciousness in the Microchips” (*cultural history*), “Ready, Set, Goo: Gooey Butter Cake is a St. Louis Classic” (*cooking*), “Bachmann Wins Iowa Straw Poll, Bests 8 GOP Contenders” (*presidential politics*), “Ten Things to Know for Friday's Championship” (*amateur sports*), “Pakistan's Constitution Avenue” (*war*), and “Morris Graves – ‘Instruments for a New Navigation’” (*style*). Illustrative examples of demarcation language from documents with high proportions of these topics include:

- “At the same time Penrose resists turning over the perplexing problems of consciousness to mysticism or other non-scientific explanations.” (*cultural history*)
- “‘Dangerously good!’ wrote one tester in our decidedly nonscientific tasting.” (*cooking*)
- “The straw poll, staged in a day-long, county-fairlike environment in Ames, Iowa, is an important if unscientific barometer.” (*presidential politics*)
- “After our highly unscientific analysis, we're giving a slight nod to Eden Prairie.” (*amateur sports*)
- “The Pakistani president vowed to reform those madrasas, or Islamic schools, that teach only the Koran, and not science, math and literature.” (*war*)
- “Their whimsical mix of pseudoscience and philosophical pretensions, their ungainly position between sculpture and painting, doesn't work well.” (*style*)

### Evaluating Topic Significance

In addition to identifying qualitatively distinct topics in a large textual dataset, computational methods provide the necessary information to evaluate the significance of topics that the model identifies. From a topic modeling perspective, there are two basic ways to think about the significance of a topic. The first is to consider how commonly a topic occurs in the corpus as a whole, relative to other topics. If a reader encounters demarcation language, what subjects are they more likely to be reading about?

**Figure 4:**
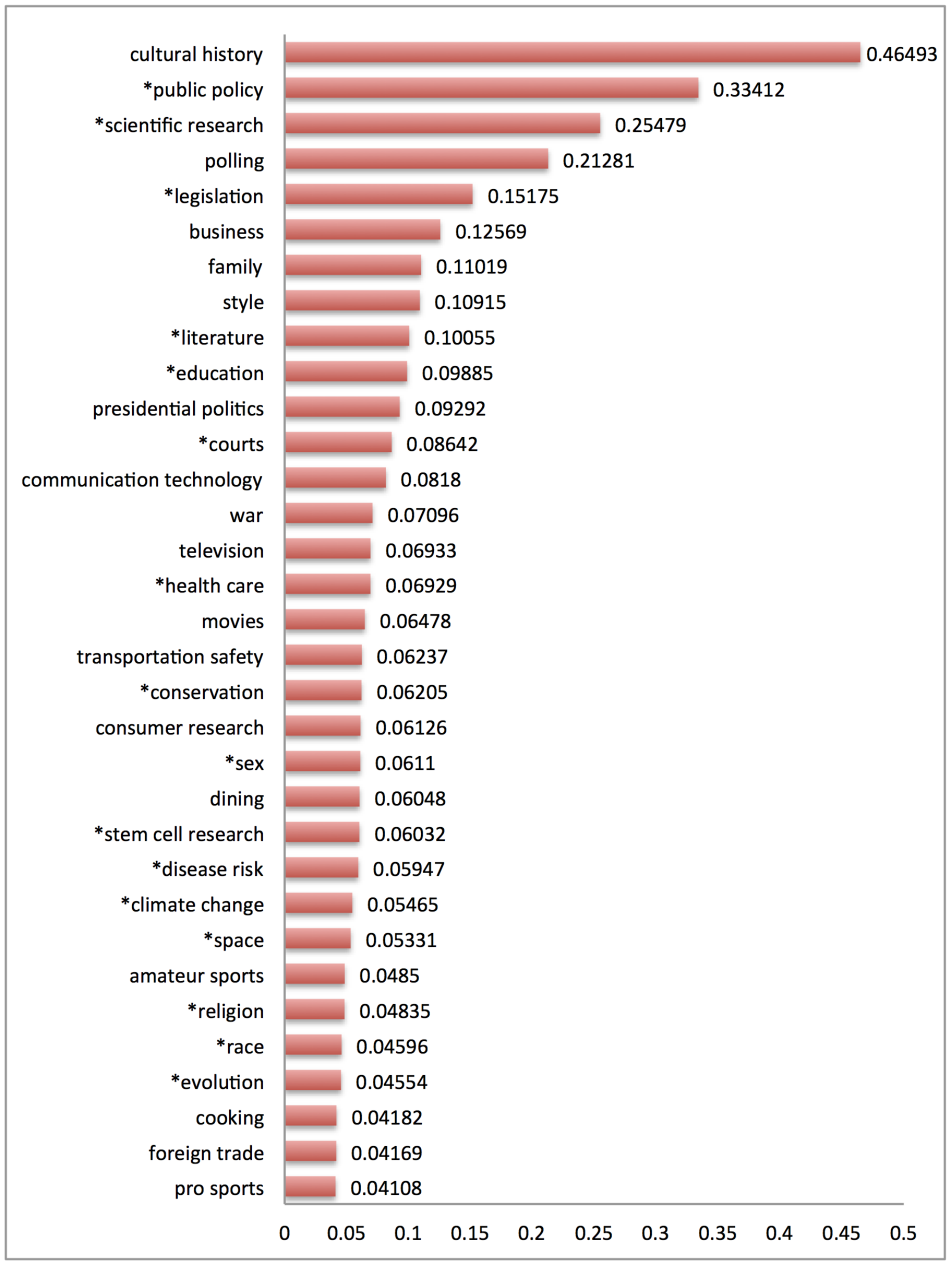
Labeled substantive topics ranked by topic Dirichlet parameter. The greater the topic Dirichlet parameter, the greater the proportion of the corpus assigned to the topic. Asterisks indicate subjects previously identified in qualitative case studies of demarcation.

Figure 4 reports labeled topics ranked by the Dirichlet parameter for each topic estimated by MALLET. In the MALLET implementation of LDA with hyperparameter optimization enabled, the Dirichlet parameter for each topic is optimized at regular intervals as the model is iteratively constructed. The greater the Dirichlet parameter for each topic in the resulting model, the greater the proportion of the corpus that has been assigned to that topic by MALLET. Ranking topics by Dirichlet parameter therefore answers the question of how commonly a topic occurs in the corpus as a whole, relative to other topics.

As Figure 4 shows, there is wide variation. Topics such as *polling* and *business* are more likely in the corpus, while topics such as *foreign trade* and *cooking* are less likely in the corpus. The interpretation would be that someone encountering an article containing demarcation language is more likely to be reading about *cultural history*, *polling* and *education*, and less likely to be reading about *evolution*, *stem cell research*, or *climate change*.

The second way to think about significance is to consider how likely it is that a topic is significant when it occurs in a document. This version is more useful for understanding the significance of a topic at the document level rather than the level of the entire corpus. If a person encounters a topic in an article from the corpus, is it usually the main topic in the article, or is it usually a minor part of the article?

**Figure 5:**
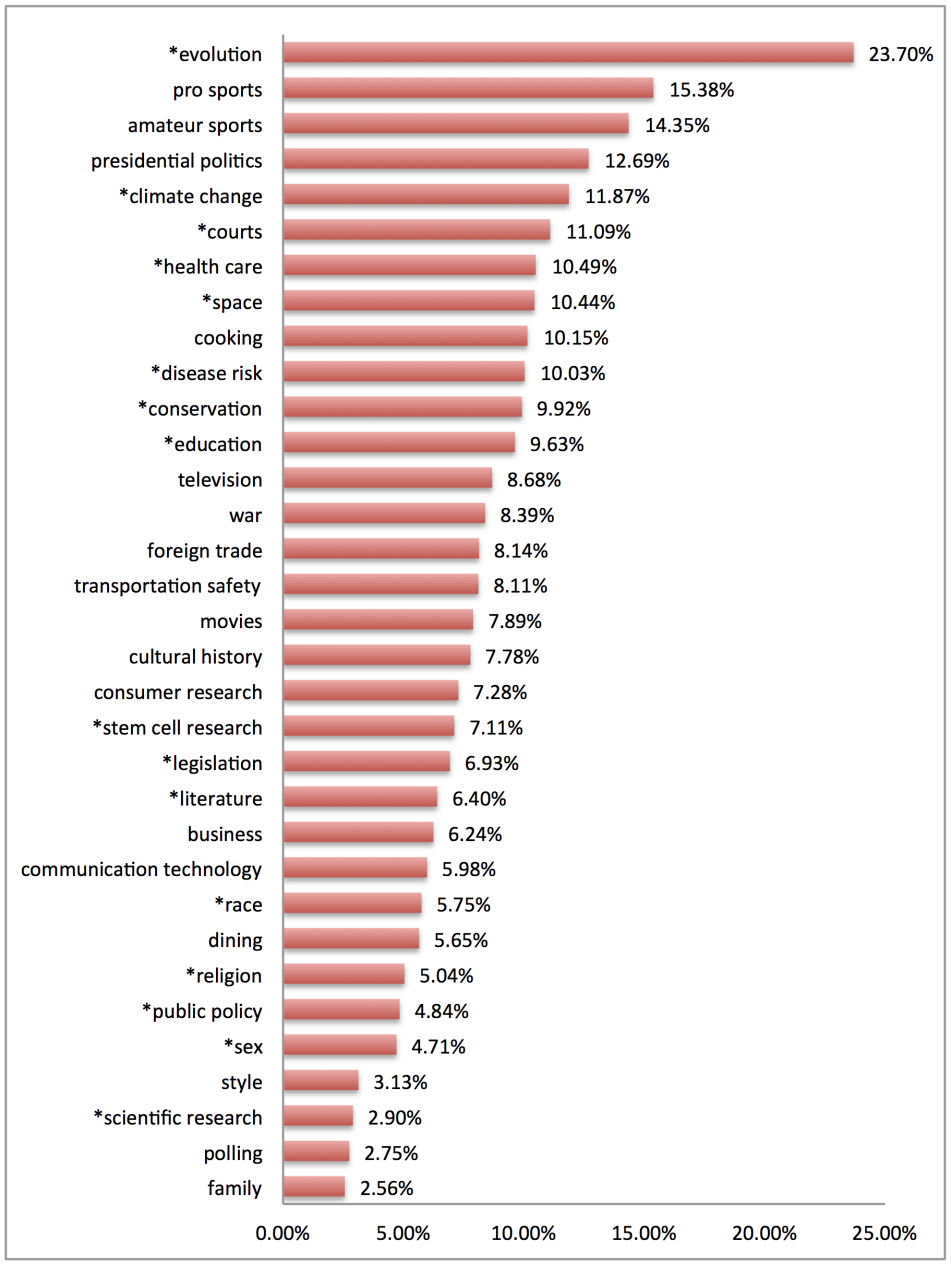
Labeled substantive topics ordered by rank_1 metric. The rank_1 metric measures the number of times a topic is the primary topic in the documents in which it occurs, here expressed as a percentage. Asterisks indicate subjects previously identified in qualitative case studies of demarcation.

Figure 5 reports labeled topics by the “rank_1” metric calculated by MALLET for each topic. (MALLET diagnostics must be enabled to generate this metric.) The rank_1 metric shows how many times each topic is ranked first in terms of document proportion (hence “rank_1”) for the documents in which it occurs. Recall that in LDA each document can be expressed as a mixture of topics. The greater the rank_1 score for a topic, the more often that topic occurs as the primary topic in a document. Ordering topics by rank_1 therefore answers the question of how significant a topic is at the document level.

As Figure 5 shows, there is wide variation again, but with a different ordering of topics. For example, while *evolution* is less significant in the overall corpus (see Figure 4), when it does appear in a document, it is the main topic in that document more than 23% of the time. A similar reranking occurs for both *pro sports* and *amateur sports*, as well as *climate change* and *space*. A reranking in the opposite direction occurs for *public policy*, *scientific research*, and *polling*, which are more significant in the overall corpus but rarely occur as the main topic in a document. Topics such as *race* and *presidential politics* remain relatively less significant or more significant, respectively, regardless of which measure of significance is used.

### Expanding Qualitative Analysis

Computational methods overcome three key limitations that case studies using small datasets encounter. First, close readings and qualitative coding approaches offer no general way of evaluating whether case studies are important or not, as small samples of textual data lack visibility to broader patterns in the public sphere. Computational methods enable analysis of much larger samples that provide the comparison data necessary to evaluate the impact of particular cases on public discourse more broadly.

**Figure 6:**
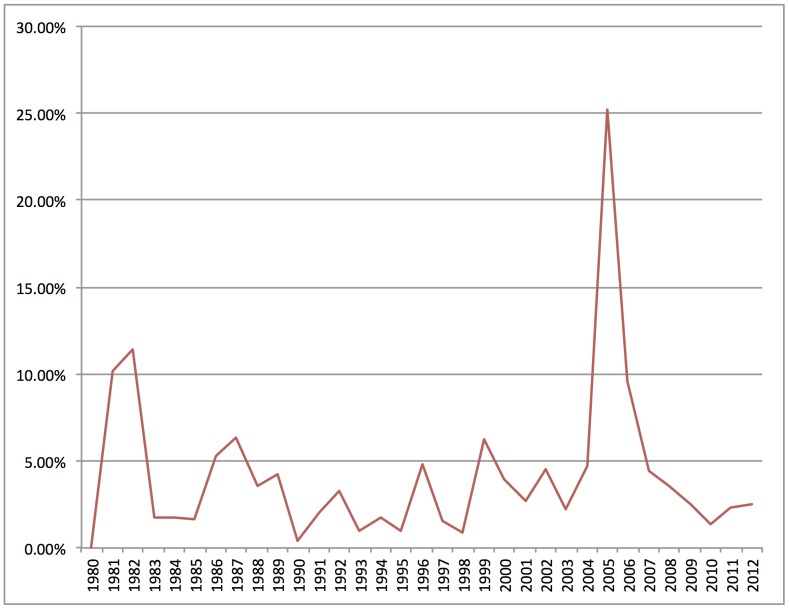
Prominence of *evolution* in demarcation corpus over time. Percentage of documents in each year with at least 15% *evolution* content.

For example, Figure 6 reports the prominence of *evolution* in the demarcation corpus over time. Many qualitative case studies have examined demarcation in public controversies over evolution, particularly in curricular challenges that result in legal battles between parents, activists, and local school boards [20]. Looking at individual documents, it is clear that the highest peaks in Figure 6, at 1982 and 2005, correspond to the two most significant court cases dealing with curricular challenges: *McLean v. Arkansas* in 1982, and *Kitzmiller v. Dover* in 2005 [31].

**Figure 7:**
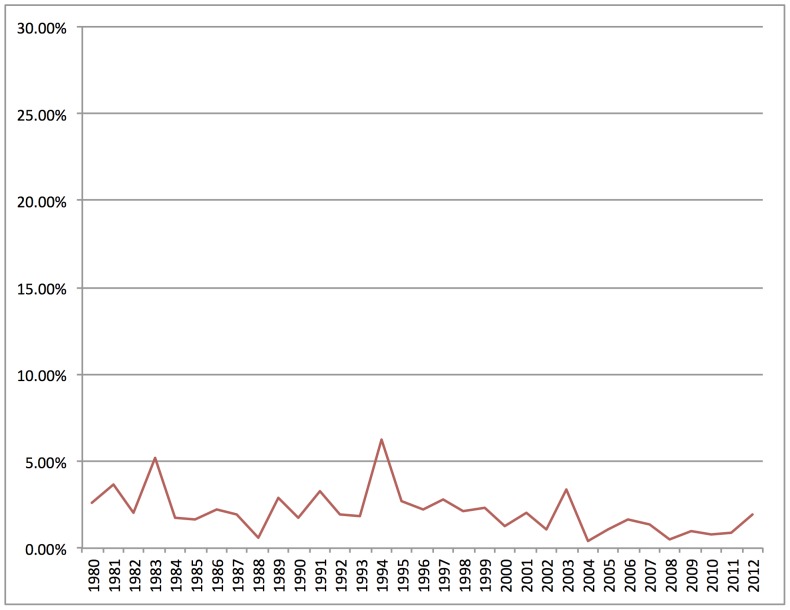
Prominence of *race* in demarcation corpus over time. Percentage of documents in each year with at least 15% *race* content.

But is demarcation activity around *evolution* more or less significant to public discourse than demarcation activity around around some other topic? Looking at thousands of documents provides the information to answer this question. As a comparison example, Figure 7 shows the prominence of *race* in the demarcation corpus over time. Like *evolution*, many qualitative case studies have examined demarcation in public controversies over race, particularly in legal battles over federal regulation of research subjects [22] and scientific disputes over genetics and IQ [32]. But unlike *evolution*, *race* not only is relatively low in prominence, but has changed very little over time.

The comparison between *evolution* and *race* in this exercise is instructive because it provides a different set of information than analysis of small textual samples on either subject can provide. Close readings and qualitative coding of selected texts related to demarcation activity around race might well point to similar strategies, features, and arguments as demarcation activity around evolution. But looking at patterns in thousands of documents reveals that activity in one case successfully constituted a large part of public demarcation discourse, while activity in another case constituted only a small part of public demarcation discourse. By providing such comparison data, computational analysis of a large textual dataset places questions about particular cases or events in broader context.

Second, and similarly, looking at thousands of documents using computational methods provides the comparison data necessary to evaluate the external validity of broader inferences drawn from close readings or qualitative coding of small samples. Take, for example, the potential use of evidence from qualitative case studies around evolution to support broader claims about the relationship between religion and science. Case studies of particular instances of controversy find consistently that such challenges have a clear religious component [31]. Looking only at these contentious instances using samples of court testimony, or newspaper coverage of specific events, it might be reasonable to infer that religion is generally in conflict with science.

**Figure 8:**
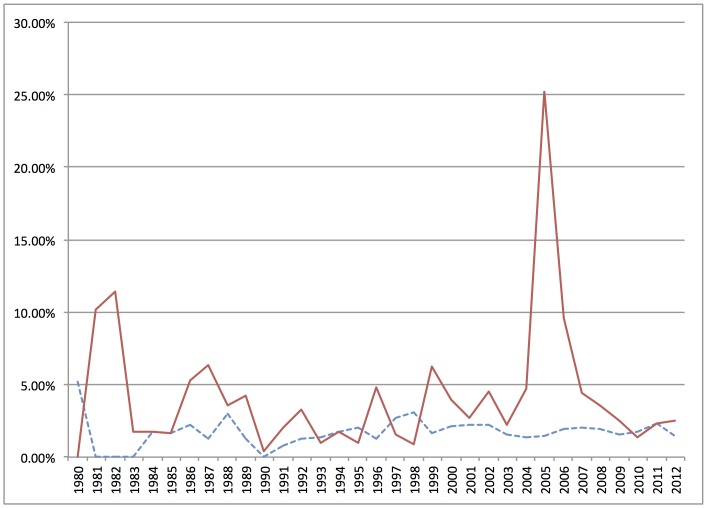
Comparison of prominence of *religion* (dotted blue) and *evolution* (solid red) in demarcation corpus over time. Percentage of documents in each year with at least 15% topic content.

But are these instances of demarcation activity involving religion exceptional or normal? Figure 8 compares the topics of *religion* and *evolution* over time. As Figure 8 shows, *religion* is not very prominent in the demarcation corpus, and it has remained not very prominent for over thirty years, while *evolution* has varied in prominence in response to specific instances of demarcation activity. In the context of the large textual dataset in this exercise, the involvement of religion in public disputes around evolution appears to be exceptional.

**Figure 9:**
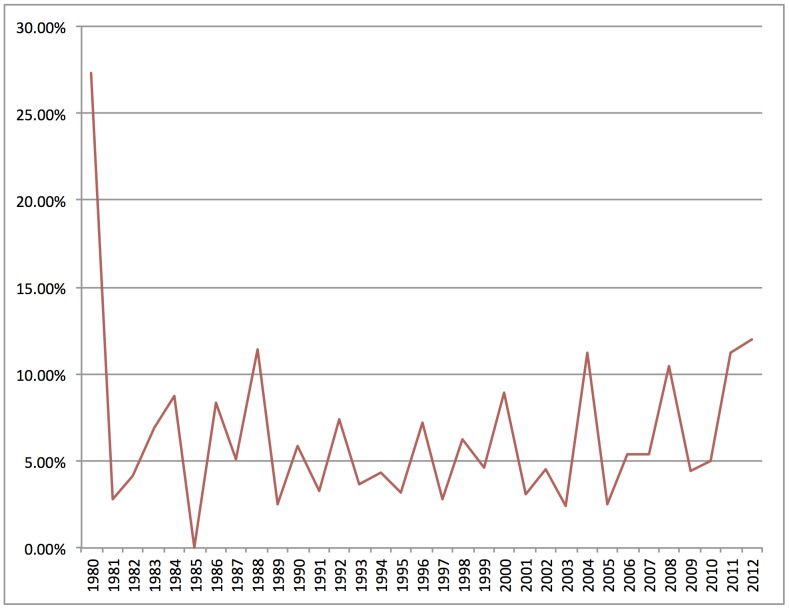
Prominence of *presidential politics* in demarcation corpus over time. Percentage of documents in each year with at least 15% *presidential politics* content.

Third, small textual samples are limited in their ability to determine whether or not particular cases are part of a consistent temporal pattern rather than unique or unusual. As an example, Figure 9 charts the prominence of *presidential politics* in the demarcation corpus over time. Figure 9 shows that more than one in four documents contained such content in 1980. Looking at individual documents, it is clear that in 1980, public conversations describing election prediction methods as (e.g.) “unscientific” or “not scientific” surged as commentators attempted to explain the unanticipated Reagan landslide win.

But is demarcation activity around *presidential politics* confined to a unique event such as the Reagan landslide, or does it recur periodically? A case study of selected documents from public discourse on the 1980 election might be able to identify the demarcation activity. But it would be unable to determine whether or not the event was unique, or to what extent it recurred in later years. Figure 9 shows that, while the topic has not returned to the heights of 1980, peaks of the distribution correspond generally to presidential election years, with the same sorts of conversations about election prediction methods recurring regularly. Where a small sample might highlight the uniqueness of public demarcation around *presidential politics*, looking at thousands of documents shows that such demarcation occurs in a regular temporal pattern and can even be anticipated in future presidential election years.

## Conclusions

Computational analysis of large textual datasets using topic modeling offers a productive way to expand qualitative research beyond the limitations of small datasets. Topic discovery does not replace close reading or qualitative coding. But, as this exercise shows, looking at thousands of documents across a broad range of subjects offers insights that are simply not available from approaches that rely on a narrow range or small set of textual data.

Computational analysis of large datasets using topic modeling also expands the scope of qualitative research by locating qualitative case studies within a broader context. Such information can be used to evaluate and compare the importance of specific case studies, to validate inferences that are drawn from qualitative analysis of small textual samples, and to determine whether specific cases are part of a consistent temporal pattern. For scholars, these additional capabilities are crucial for placing qualitative findings in the context of broader theoretical and empirical problems.

For all of its benefits, topic modeling of large datasets involves three important limitations. First, this approach does not address questions about who is using this language. In the demarcation example, who is making these distinctions, and why? Second, this approach does not address the target of language use. In the demarcation example, is the target of demarcation a person, an idea, or a set of activities? Third, this approach does not address why one form of language rather than another might be deployed. In the demarcation example, why use “anti-science” rather than “pseudoscience,” “non-science,” or “unscientific” in making distinctions?

Such questions cannot be addressed only with topic modeling. Future research should employ more conventional qualitative methods to pursue these lines of inquiry. But topic modeling generatively extends the reach of qualitative analysis beyond its current limits, and points to productive new directions for exploration.
